# Effect of Ovarian Stimulation and Trigger Protocols on Oocyte and Embryo Numbers—Real World Experience

**DOI:** 10.3390/jcm14176096

**Published:** 2025-08-28

**Authors:** Shmuel Somer, Simon Nothman, Shira Baram, Ido Izhaki, Nitzan Dana Sela, Ronit Beck-Fruchter

**Affiliations:** 1 Sheba Medical Center, Tel Hashomer, Ramat Gan 52621, Israel; 2 Genea Fertility, Sydney, NSW 2000, Australia; connect@drsimonnothman.com.au; 3 Fertility and In-Vitro Fertilization Unit, Department of Obstetrics and Gynecology, Emek Medical Center, Afula 1834111, Israel; shira_ba@clalit.org.il (S.B.); nitzanse@clalit.org.il (N.D.S.); ronitbeck@gmail.com (R.B.-F.); 4 Rappaport Faculty of Medicine, Technion Institute of Technology, Haifa 3525433, Israel; 5 Department of Evolutionary and Environmental Biology, Faculty of Natural Sciences, University of Haifa, Haifa 3103301, Israel; izhaki@research.haifa.ac.il

**Keywords:** double trigger, dual trigger, IVF, trigger protocols, oocyte numbers, embryo numbers

## Abstract

**Objectives**: This retrospective single-center cohort study aims to evaluate the impact of dual-trigger therapy (recombinant hCG [rhCG] combined with GnRH agonist) compared to rhCG alone on ART outcomes in women undergoing GnRH antagonist protocols. **Methods**: Data from 1291 IVF cycles performed between 2016 and 2022 were analyzed. After propensity score matching (PSM) to adjust for confounders, 395 cycles in each group were compared. Primary outcomes included the total number of oocytes retrieved, while secondary outcomes assessed mature oocytes, fertilization rates, and embryo numbers. **Results**: Dual-trigger therapy yielded significantly more total oocytes (7.50 ± 5.23 vs. 6.12 ± 4.23, *p* < 0.001) and mature oocytes (5.67 ± 3.87 vs. 5.01 ± 3.13, *p* = 0.047) compared to rhCG alone. Cycles with no oocytes were fewer in the dual-trigger group (1.3% vs. 3.8%, *p* = 0.015). Total embryos were also higher with dual trigger therapy (2.43 ± 1.90 vs. 2.00 ± 1.93, *p* = 0.001). In intracytoplasmic sperm injection (ICSI) cycles, the fertilization rate significantly improved with dual trigger (64.93 ± 33.50% vs. 52.22 ± 34.12%, *p* = 0.003). No significant differences were noted in fertilization rates for standard IVF (55.14 ± 30.72% vs. 52.29 ± 32.11%, *p* = 0.18) or maturation rates (72.52 ± 26.91% vs. 71.53 ± 24.75%, *p* = 0.37). **Conclusions**: These findings demonstrate that dual-trigger therapy improves ART outcomes by increasing oocyte and embryo yields.

## 1. Introduction

The first in vitro fertilization (IVF) child, Louise Brown, was conceived following oocyte aspiration shortly after the onset of the mid-cycle luteinizing hormone (LH) surge during a natural cycle [1]. For decades thereafter, human chorionic gonadotropin (hCG) has been used to successfully trigger final oocyte maturation and ovulation, acting as a substitute for the endogenous LH surge due to its similar mechanism of action [2].

In the 1990s, early studies examined the use of a GnRH agonist bolus as an alternative to hCG, finding it to be a more physiological trigger with favorable outcomes [3,4]. During this period, GnRH agonist-based down-regulation protocols were routinely employed in most assisted reproductive technology (ART) cycles, which rendered the pituitary unresponsive, preventing an endogenous LH surge. As a result, the application of the GnRH agonist as an ovulation trigger was limited [5]. With the introduction of GnRH antagonist protocols, the use of the GnRH agonist trigger became viable once again. However, despite the successful retrieval of mature oocytes and the development of high-quality embryos—along with a significant reduction in the risk of ovarian hyperstimulation syndrome—the clinical outcomes were disappointing, with high rates of early pregnancy loss and lower ongoing pregnancy rates [6].

The initial studies proposing the dual trigger approach were highlighted in several key articles. Kol et al. suggested that a dual-trigger method could provide maximal stimulation for oocyte maturation, potentially improving clinical outcomes [7]. Lin et al. demonstrated that using a dual trigger in GnRH antagonist cycles for normal responders significantly improves implantation, clinical pregnancy, and live birth rates, providing further clinical validation for this approach [8]. Finally, Orvieto reviewed the various trigger strategies, summarizing the existing evidence [9]. These studies formed the foundation for understanding the potential of dual-trigger strategies in women at low risk for OHSS, offering a promising path to improved clinical outcomes in fertility treatments.

Although dual trigger therapy has been increasingly used in various clinical situations and has shown many potential benefits [10], it remains controversial whether the dual trigger applies to all types of patients [11]. In this study, we aim to compare the effectiveness of the dual trigger with the single hCG trigger in improving the number of oocytes retrieved, the number of matured oocytes, and the number of embryos.

Additionally, the timing of the trigger concerning the GnRH antagonist should be studied. There is a paucity of data on this topic dating back to 1992, when Donoghue et al. performed a study in cats assessing the influence of gonadotropic treatment intervals on follicular maturation [12]. They showed that the timing of the hormonal stimulus can impact oocyte production and maturation [12]. In 2000, Itskovitz et al. demonstrated that hypophyseal sensitivity resumed 30 h after discontinuing GnRH antagonist treatment [5]. Alternatively, Horowitz et al. showed that shorter intervals between stopping antagonist therapy and starting an agonist trigger did not negatively affect outcomes [13]. The appropriate interval between antagonist cessation and agonist administration remains an unresolved topic in reproductive medicine.

This study assesses the impact of modifying our treatment protocol for normal responders by transitioning from an hCG trigger to a dual trigger. Additionally, gonadotropin and GnRH antagonist treatments were extended up to the trigger day. The evaluation focused on key outcome measures, including the total number of retrieved oocytes, the count of mature oocytes, and the number of viable embryos. This approach aims to determine if these changes can enhance overall treatment success and improve clinical results.

## 2. Materials and Methods

### 2.1. Study Design and Participants

This was a retrospective single-center cohort study of women who underwent ART treatment in GnRH antagonist protocol from 2016–2022. We accessed the database, as detailed below, of routinely collected data for all IVF cycles recording patients, treatments, and outcome parameters at our facility, collected with the approval of our institutional ethics committee for quality control and research (0141-22).

### 2.2. Inclusion and Exclusion Criteria

In this study, we screened all ART treatment cycles performed during the study period. Only patients undergoing GnRH antagonist protocol stimulation were included. Patients who were at high risk for OHSS or were undergoing oocyte/embryo cryopreservation and thus used a single trigger of GnRH agonist were excluded. Additionally, patients whose IVF cycle was canceled, and thus had no ovum pickup, were excluded as well.

### 2.3. Treatment Protocol

In the past, patients who were at low risk for OHSS were triggered in our IVF unit by recombinant hCG (rhCG) (choriogonadotropin alfa, OVITRELLE 250 mcg/0.5 mL) alone. Since 2020, our protocol has changed and all these patients received dual trigger, with rhCG and GnRH agonist (Triptorelin, Decapeptyl 0.2 mg). Additionally, before 2020 the last gonadotropin and GnRH antagonist injections were given one day before triggering with a single shot of rhCG. After 2020, gonadotropin in addition to a GnRH antagonist was given on the day of triggering which was achieved using a rhCG concomitant with a GnRH agonist.

### 2.4. Outcome Variables

The primary outcome measures were the number of oocytes. The secondary outcomes included the number of cycles with no oocytes, the number of mature oocytes, the maturation rate, the number of fertilizations, the fertilization rate in IVF, the fertilization rate in intracytoplasmic sperm injection (ICSI), and the total number of embryos.

### 2.5. Statistical Analysis

Patient statistical information was analyzed using Microsoft Excel. Patient demographics and history were included in addition to detailed ART protocols and information. Patient parameters were assessed via t-test or Mann–Whitney U test in the case of non-normally distributed data for continuous data or χ2 or Fisher’s exact test where it was appropriate for categorical data. A 1:1 propensity score matching (PSM) was performed to adjust for confounding effects using SAS/STAT software version 9.4 (Cary, NC, USA, 2023). Maternal age, body mass index (BMI), basal FSH level, infertility duration, gravity, parity, and number of previous cycles were included in the PSM model.

## 3. Results

**Figure 1:**
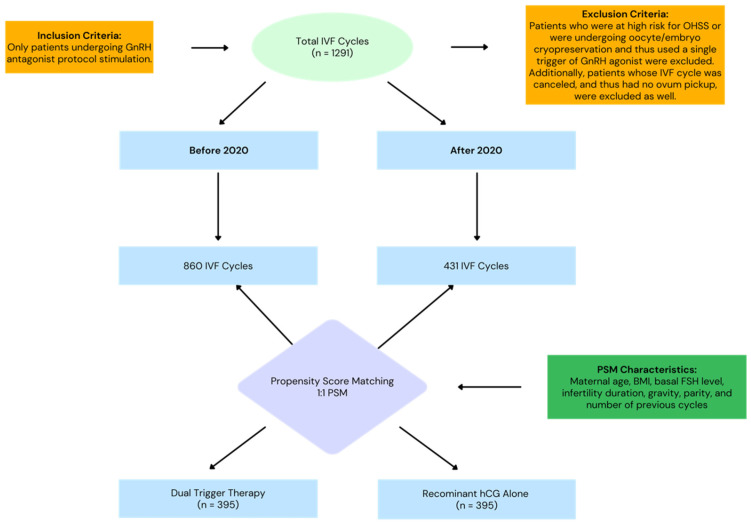
Study Flow Chart—The study analyzed a total of 907 IVF cycles split between the periods before 2020 and after 2020. Before 2020 there were 631 IVF cycles performed and after there were 276 performed. Propensity score matching led us to compare 395 cycles in each group. We compared dual trigger therapy with recombinant hCG alone. The study included only those undergoing GnRH antagonist protocol stimulation. It excluded anyone at high risk for OHSS or who was undergoing oocyte/embryo cryopreservation and thus used a single trigger of GnRH agonist. Additionally, we excluded patients whose IVF cycle was canceled and thus had no ovum pickup.

The study analyzed a total of 1291 treatment cycles. Of these, 860 cycles were conducted in the years 2016–2020, while 431 cycles were carried out after 2020. Subsequently, 395 cycles were matched in a 1:1 ratio after PSM (Figure 1).

After adjusting baseline characteristics using PSM, two groups of 395 patients each were analyzed. The control sample included 395 patients who were treated with rHCG only and withheld antagonists and gonadotropins 30 h before the trigger (control). The study sample consisted of 395 patients who received dual trigger therapy and received gonadotropins and GnRH antagonists on the day of the trigger (study). Propensity score matching for baseline characteristics showed no significant baseline differences in age, BMI, FSH, number of previous cycles, years of infertility, gravidity, or parity (Table 1).

The primary outcome showed significant differences between the control and study groups. The control group had a total number of 2417 oocytes among its patients, while the study group had a total number of 2962 oocytes. This equates to the average number of oocytes being 6.12 ± 4.23 and 7.50 ± 5.23 in the control and study groups, respectively (*p* < 0.001).

The secondary outcomes included the number of cycles with no oocytes, the number of mature oocytes, the maturation rate, the number of fertilizations (2PN), and the fertilization rate in IVF and ICSI. The study group had a significantly lower number of cycles with no oocytes, significantly more mature oocytes, a significant difference in the number of fertilizations, a significantly higher fertilization rate of eggs in ICSI, and a significantly higher number of total embryos compared to the control group (Table 2).

## 4. Discussion

In the present study, we compared the co-administration of GnRHa and rhCG as a dual trigger with a single rhCG trigger in women undergoing ART treatment. Our study showed significantly improved outcomes in the dual trigger therapy compared to the rhCG therapy in the total number of oocytes. It additionally showed a significantly higher fertilization rate of eggs in ICSI, a higher number of total embryos, more mature oocytes, and a higher number of fertilizations. Our study additionally showed a significantly lower number of cycles with no oocytes.

The traditional standard for ovulation induction has been rhCG, acting as a surrogate for the mid-cycle LH surge by binding to the LH/hCG receptor [14]. However, due to its prolonged half-life (~24 h compared to ~1 h for LH), hCG can lead to sustained luteotropic activity and an increased risk of OHSS [15]. Conversely, GnRH agonist triggers induce both an LH and FSH surge, resembling ovulation’s natural hormonal environment [16].

The evidence regarding the exact outcomes of dual trigger protocols in assisted reproductive technology (ART) remains controversial, as different studies report conflicting results. This discrepancy between studies highlights the ongoing debate regarding the clinical benefits of the dual trigger protocol, particularly in terms of optimizing oocyte retrieval. Our study aims to contribute to the literature and add real-world data.

A single-center, randomized-controlled, double-blinded clinical trial on a small sample size of 155 patients demonstrated significantly higher pregnancy and live birth rates per transfer in the dual trigger group compared to the hCG-only group [16]. In contrast, an open-label randomized controlled trial found no significant differences in pregnancy outcomes from both fresh and frozen embryo transfers [17]. Furthermore, a 2024 meta-analysis conducted by Beebeejaun et al. found no evidence supporting improved clinical pregnancy rates [15].

An open-label randomized controlled trial reported a higher retrieval rate and an increase in the number of good-quality and viable embryos in the dual trigger group. However, this study did not show a significant increase in the number of retrieved oocytes or the rates of 2 pronuclear (2PN) embryos [17].

A retrospective cohort study showed no significant differences in the number of oocytes retrieved, embryos available, or the number of top-quality embryos. It showed a significantly higher rate of miscarriages in the dual trigger group compared to the hCG alone [18]. A retrospective study showed a slight increase in the number of oocytes and embryos in the dual trigger group. It did not, however, show a difference in live births [19].

Our outcomes showed a significantly increased number of oocytes and the number of embryos in the study group compared to the control group. This has previously been proven to lead to increased ART success rates. We also showed a significantly lower number of cycles with no oocytes, a significant increase in the number of mature oocytes, and fertilizations, and a significantly higher fertilization rate in ICSI. The maturation rate, however, was not increased. This proves that the increased number of mature oocytes is due to the increased number of oocytes retrieved and does not increase the maturation rate.

There were more fertilized eggs in the dual-trigger group. When looking at the percentage of fertilized eggs, no statistically significant difference was found in the IVF group, but there was in the ICSI group. We hypothesize that the dual trigger, being more similar to the physiological trigger, not only increases the number of retrieved eggs but also improves their quality, which is reflected in the increase in the percentage of fertilized eggs. The increase in the number of retrieved eggs, mature eggs, and fertilized eggs ultimately increases the number of embryos, which is expected to be related to an improvement in the success rate of the treatment [20].

The strength of our study is that we used PSM analysis to control for potential confounders between the two groups, thus making the outcomes independent from the different baseline characteristics. Moreover, because of the single-center design of this study, all ART cycles were carried out under uniform protocol and same laboratory conditions thus limiting biases.

Real-world data provides distinct advantages, even when sourced from retrospective studies. It captures the complexities and variabilities inherent in clinical practice, enabling a more comprehensive understanding of how interventions perform across diverse patient populations. This broader perspective aids in informing treatment strategies and enhancing patient care in real-world settings. In line with the real-world nature of this study, certain clinically relevant variables such as OHSS incidence, embryo quality, number of fresh transfers, and pregnancy outcomes were not consistently captured in our retrospective dataset and were therefore not included in the analysis. These elements may further inform the impact of dual trigger protocols and warrant prospective evaluation in future studies.

The improved outcomes seen with dual trigger therapy may be attributed to its ability to better mimic the natural mid-cycle hormonal environment. The GnRH agonist induces an endogenous surge of both LH and FSH, whereas rhCG mimics only LH. The FSH surge triggered by the GnRH agonist is believed to support cumulus expansion, oocyte detachment, and nuclear maturation. Meanwhile, the addition of rhCG provides a sustained LH-like effect, supporting final oocyte maturation and luteal phase support. This synergistic hormonal activity likely contributes to the increased number of mature oocytes and embryos observed with dual triggering [7,8].

Although dual trigger therapy demonstrated statistically significant improvements in oocyte and embryo yield, the clinical relevance of this increase may vary depending on patient goals and resource availability. The addition of a GnRH agonist introduces minimal cost, but in settings where cost-effectiveness is paramount, the marginal benefit must be weighed carefully. For patients seeking cumulative embryo yield for multiple transfers or fertility preservation, even a modest increase may be impactful. However, in cases where only a single embryo transfer is intended, the benefit may be less pronounced.

However, this study has some limitations including the retrospective design as the main one. Alongside the alteration of the trigger protocol, another modification was implemented. Prior to 2020, patients received the final gonadotropin and GnRH antagonist injections the day before triggering, approximately 30 h before administering rhCG. After 2020, patients were instructed to receive the final gonadotropin and antagonist injections on the same day as triggering.

Although there are concerns that administering the GnRH antagonist shortly before the agonist may diminish the effectiveness of the agonist trigger, we believe this change does not adversely affect the significance of our results. In a study in 2020, Horowitz et al. concluded that the time interval between the last antagonist dose and the agonist trigger does not influence the success rates of an effective LH surge and of oocyte maturation and release [13].

Propensity scores reduce the sample size, increasing the risk of type II errors. There may still be potential variables that were not considered in the regression analysis, which could also contribute to biased results. Hence, future studies should employ an RCT design in a larger sample size to verify and validate our results.

## 5. Conclusions

Dual trigger therapy was associated with significantly improved oocyte and embryo yields and may represent a valuable tool for enhancing ART outcomes. These results suggest that, when appropriately tailored to the patient’s clinical profile, dual triggering has the potential to benefit a wide range of patients, particularly normo-responders seeking to optimize embryo numbers, while encouraging further exploration of its role across diverse populations.

## Figures and Tables

**Table 1:** Baseline Characteristics after PSM.

Baseline	rhCG *n* = 395	rhCG + GnRH Agonist *n* = 395	*p*-Value
Age	35.68 + 5.85	35.58 ± 6.00	*p* = 0.82
BMI	27.20 ± 6.03	27.35 ± 5.91	*p* = 0.73
FSH	1.47 ± 1.15	1.39 ± 1.15	*p* = 0.35
Number of Previous Cycles	1.81 ± 2.42	1.89 ± 2.71	*p* = 0.69
Years of Infertility	1.63 ± 1.10	1.63 ± 1.09	*p* = 1.0
Gravidity	1.42 ± 1.72	1.18 ± 1.64	*p* = 0.052
Parity	0.58 ± 0.72	0.54 ± 0.69	*p* = 0.44

**Table 2:** Comparison of Outcomes Between Trigger Groups.

Results	rhCG *n* = 395	rhCG + GnRH Agonist *n* = 395	*p*-Value
Total Number of Oocytes	*n* = 2417	*n* = 2962	***p* < 0.001**
Average Number of Oocytes	6.12 ± 4.23	7.50 ± 5.23	***p* < 0.001**
Number of Cycles with no Oocytes	15/395 (3.8%)	5/382 (1.3%)	***p* = 0.015**
Number of Mature Oocytes	5.01 ± 3.13	5.67 ± 3.87	***p* = 0.047**
Maturation Rate	71.53 ± 24.75	72.52 ± 26.91	*p* = 0.37
Number of Fertilizations (2PN)	2.92 ± 2.97	3.73 ± 3.30	***p* < 0.001**
Fertilization Rate in IVF	52.29 ± 32.11	55.14 ± 30.72	*p* = 0.18
Fertilization Rate in ICSI	52.22 ± 34.12	64.93 ± 33.5	***p* = 0.003**
Total Number of Embryos	2.00 ± 1.93	2.43 ± 1.90	***p* = 0.001**

## Data Availability

This study is based on a retrospective single-center cohort analysis. The dataset analyzed during this study is available upon reasonable request, as per institutional policies and ethical guidelines.

## Abbreviations

The following abbreviations are used in this manuscript:

ART	Assisted Reproductive Technology
BMI	Body Mass Index
FSH	Follicle Stimulating Hormone
GnRH	Gonadotropin-Releasing Hormone
hCG	Human Chorionic Gonadotropin
ICSI	Intracytoplasmic Sperm Injection
IRB	Institutional Review Board
IVF	In Vitro Fertilization
OHSS	Ovarian Hyperstimulation Syndrome
PSM	Propensity Score Matching
2PN	2 Pronuclei
